# Supplementary material for: Role and mechanism of NCAPD3 in promoting malignant behaviors in gastric cancer
Source: Front Pharmacol. 2024 Apr 22;15:1341039. doi: 10.3389/fphar.2024.1341039 (PMC11070777; doi:10.3389/fphar.2024.1341039)
Supplement: Supplementary file 11 [file DataSheet2.ZIP › GSEA/Transcipt factor/my_analysis.Gsea.1599461814734/ARID5B_TARGET_GENES.html]

Details for gene set ARID5B\_TARGET\_GENES[GSEA]

|  || Dataset | filtered\_dataset.sample\_info.cls#WT\_versus\_NCAPD3\_MUT |
| Phenotype | sample\_info.cls#WT\_versus\_NCAPD3\_MUT |
| Upregulated in class | WT |
| GeneSet | ARID5B\_TARGET\_GENES |
| Enrichment Score (ES) | 0.287012 |
| Normalized Enrichment Score (NES) | 1.9636396 |
| Nominal p-value | 0.0 |
| FDR q-value | 0.16884731 |
| FWER p-Value | 0.724 |
Table: GSEA Results Summary

  

Fig 1: Enrichment plot: ARID5B\_TARGET\_GENES      
 Profile of the Running ES Score & Positions of GeneSet Members on the Rank Ordered List

  

| SYMBOL | TITLE | RANK IN GENE LIST | RANK METRIC SCORE | RUNNING ES | CORE ENRICHMENT || 1 | 51111 | KMT5B | 2 | 1.217 | 0.0223 | Yes |
| 2 | 2935 | GSPT1 | 5 | 1.156 | 0.0434 | Yes |
| 3 | 51351 | ZNF117 | 14 | 1.052 | 0.0579 | Yes |
| 4 | 55149 | MTPAP | 15 | 1.051 | 0.0785 | Yes |
| 5 | 55727 | BTBD7 | 38 | 0.954 | 0.0805 | Yes |
| 6 | 80746 | TSEN2 | 53 | 0.909 | 0.0876 | Yes |
| 7 | 23322 | RPGRIP1L | 60 | 0.871 | 0.1001 | Yes |
| 8 | 157680 | VPS13B | 78 | 0.838 | 0.1036 | Yes |
| 9 | 50861 | STMN3 | 126 | 0.777 | 0.0831 | Yes |
| 10 | 6925 | TCF4 | 132 | 0.768 | 0.0944 | Yes |
| 11 | 51174 | TUBD1 | 153 | 0.730 | 0.0934 | Yes |
| 12 | 9848 | MFAP3L | 155 | 0.726 | 0.1069 | Yes |
| 13 | 7248 | TSC1 | 158 | 0.718 | 0.1195 | Yes |
| 14 | 10206 | TRIM13 | 161 | 0.716 | 0.1320 | Yes |
| 15 | 54815 | GATAD2A | 191 | 0.685 | 0.1233 | Yes |
| 16 | 4836 | NMT1 | 208 | 0.669 | 0.1243 | Yes |
| 17 | 85407 | NKD1 | 228 | 0.652 | 0.1226 | Yes |
| 18 | 55745 | AP5M1 | 246 | 0.634 | 0.1221 | Yes |
| 19 | 9778 | KIAA0232 | 247 | 0.633 | 0.1345 | Yes |
| 20 | 10944 | C11orf58 | 272 | 0.617 | 0.1283 | Yes |
| 21 | 199857 | ALG14 | 275 | 0.615 | 0.1389 | Yes |
| 22 | 55275 | VPS53 | 278 | 0.609 | 0.1493 | Yes |
| 23 | 80124 | VCPIP1 | 282 | 0.607 | 0.1589 | Yes |
| 24 | 10585 | POMT1 | 284 | 0.606 | 0.1700 | Yes |
| 25 | 84078 | KBTBD7 | 295 | 0.594 | 0.1740 | Yes |
| 26 | 81889 | FAHD1 | 299 | 0.592 | 0.1833 | Yes |
| 27 | 1021 | CDK6 | 324 | 0.579 | 0.1764 | Yes |
| 28 | 4297 | KMT2A | 337 | 0.573 | 0.1785 | Yes |
| 29 | 2342 | FNTB | 344 | 0.570 | 0.1851 | Yes |
| 30 | 9315 | NREP | 364 | 0.557 | 0.1816 | Yes |
| 31 | 5036 | PA2G4 | 365 | 0.557 | 0.1925 | Yes |
| 32 | 1080 | CFTR | 366 | 0.557 | 0.2034 | Yes |
| 33 | 57109 | REXO4 | 370 | 0.552 | 0.2119 | Yes |
| 34 | 805 | CALM2 | 423 | 0.518 | 0.1826 | Yes |
| 35 | 801 | CALM1 | 424 | 0.518 | 0.1927 | Yes |
| 36 | 54665 | RSBN1 | 429 | 0.516 | 0.1998 | Yes |
| 37 | 22796 | COG2 | 445 | 0.507 | 0.1983 | Yes |
| 38 | 200933 | FBXO45 | 456 | 0.500 | 0.2005 | Yes |
| 39 | 5295 | PIK3R1 | 467 | 0.493 | 0.2026 | Yes |
| 40 | 79960 | JADE1 | 468 | 0.493 | 0.2122 | Yes |
| 41 | 146691 | TOM1L2 | 472 | 0.490 | 0.2195 | Yes |
| 42 | 7109 | TRAPPC10 | 475 | 0.488 | 0.2276 | Yes |
| 43 | 55705 | IPO9 | 476 | 0.487 | 0.2371 | Yes |
| 44 | 5245 | PHB | 481 | 0.486 | 0.2436 | Yes |
| 45 | 5188 | GATB | 512 | 0.469 | 0.2300 | Yes |
| 46 | 29090 | TIMM21 | 537 | 0.453 | 0.2206 | Yes |
| 47 | 3609 | ILF3 | 544 | 0.449 | 0.2248 | Yes |
| 48 | 9836 | LCMT2 | 549 | 0.448 | 0.2306 | Yes |
| 49 | 1819 | DRG2 | 550 | 0.447 | 0.2393 | Yes |
| 50 | 9416 | DDX23 | 553 | 0.445 | 0.2465 | Yes |
| 51 | 7112 | TMPO | 566 | 0.439 | 0.2460 | Yes |
| 52 | 55125 | CEP192 | 568 | 0.438 | 0.2538 | Yes |
| 53 | 5198 | PFAS | 570 | 0.437 | 0.2616 | Yes |
| 54 | 55245 | UQCC1 | 580 | 0.434 | 0.2633 | Yes |
| 55 | 8021 | NUP214 | 611 | 0.417 | 0.2487 | Yes |
| 56 | 84948 | TIGD5 | 619 | 0.413 | 0.2514 | Yes |
| 57 | 10061 | ABCF2 | 626 | 0.408 | 0.2549 | Yes |
| 58 | 54663 | WDR74 | 628 | 0.408 | 0.2621 | Yes |
| 59 | 9852 | EPM2AIP1 | 634 | 0.406 | 0.2662 | Yes |
| 60 | 11226 | GALNT6 | 659 | 0.397 | 0.2558 | Yes |
| 61 | 51287 | COA4 | 660 | 0.396 | 0.2635 | Yes |
| 62 | 23250 | ATP11A | 664 | 0.395 | 0.2690 | Yes |
| 63 | 7587 | ZNF37A | 667 | 0.394 | 0.2752 | Yes |
| 64 | 57493 | HEG1 | 683 | 0.386 | 0.2714 | Yes |
| 65 | 157570 | ESCO2 | 685 | 0.386 | 0.2782 | Yes |
| 66 | 285636 | C5orf51 | 692 | 0.382 | 0.2811 | Yes |
| 67 | 5987 | TRIM27 | 695 | 0.380 | 0.2870 | Yes |
| 68 | 51000 | SLC35B3 | 740 | 0.355 | 0.2605 | No |
| 69 | 102288414 | C11orf98 | 785 | 0.330 | 0.2336 | No |
| 70 | 79590 | MRPL24 | 800 | 0.320 | 0.2292 | No |
| 71 | 516 | ATP5G1 | 826 | 0.301 | 0.2161 | No |
| 72 | 253725 | FAM21C | 829 | 0.297 | 0.2204 | No |
| 73 | 2224 | FDPS | 841 | 0.273 | 0.2174 | No |
| 74 | 7357 | UGCG | 852 | -0.254 | 0.2148 | No |
| 75 | 54968 | TMEM70 | 879 | -0.291 | 0.2007 | No |
| 76 | 990 | CDC6 | 888 | -0.304 | 0.2006 | No |
| 77 | 27230 | SERP1 | 892 | -0.306 | 0.2043 | No |
| 78 | 4209 | MEF2D | 910 | -0.337 | 0.1980 | No |
| 79 | 4088 | SMAD3 | 930 | -0.353 | 0.1905 | No |
| 80 | 8881 | CDC16 | 963 | -0.383 | 0.1736 | No |
| 81 | 9572 | NR1D1 | 977 | -0.392 | 0.1714 | No |
| 82 | 84919 | PPP1R15B | 979 | -0.394 | 0.1784 | No |
| 83 | 51343 | FZR1 | 1005 | -0.407 | 0.1674 | No |
| 84 | 51646 | YPEL5 | 1014 | -0.413 | 0.1694 | No |
| 85 | 81631 | MAP1LC3B | 1060 | -0.452 | 0.1440 | No |
| 86 | 100534599 | ISY1-RAB43 | 1082 | -0.462 | 0.1371 | No |
| 87 | 10098 | TSPAN5 | 1112 | -0.485 | 0.1246 | No |
| 88 | 1032 | CDKN2D | 1156 | -0.519 | 0.1021 | No |
| 89 | 639 | PRDM1 | 1159 | -0.523 | 0.1108 | No |
| 90 | 54407 | SLC38A2 | 1182 | -0.553 | 0.1049 | No |
| 91 | 10001 | MED6 | 1190 | -0.561 | 0.1106 | No |
| 92 | 22809 | ATF5 | 1246 | -0.624 | 0.0810 | No |
| 93 | 9262 | STK17B | 1268 | -0.659 | 0.0780 | No |
| 94 | 91947 | ARRDC4 | 1282 | -0.671 | 0.0812 | No |
| 95 | 1649 | DDIT3 | 1341 | -0.779 | 0.0524 | No |
Table: GSEA details [plain text format]

  

Fig 2: ARID5B\_TARGET\_GENES      
 Blue-Pink O' Gram in the Space of the Analyzed GeneSet

  

Fig 3: ARID5B\_TARGET\_GENES: Random ES distribution      
 Gene set null distribution of ES for **ARID5B\_TARGET\_GENES**
